# Highly Educated Migrants in Norway: Identity and Well-Being During Delayed Workforce Entry—A Qualitative Study

**DOI:** 10.3390/ijerph23070896

**Published:** 2026-07-12

**Authors:** Siv Karin Eriksen, Khadra Yasien Ahmed, Esperanza Diaz, Astrid Blystad

**Affiliations:** 1 Department of Global Public Health and Primary Care, University of Bergen, 5007 Bergen, Norway; eriksen.siv.k@gmail.com (S.K.E.); esperanza.diaz@uib.no (E.D.); astrid.blystad@uib.no (A.B.); 2 Centre for International Health, Department of Global Public Health and Primary Care, University of Bergen, 5007 Bergen, Norway

**Keywords:** migrants, health, work, meaningful work, integration, social identity, well-being

## Abstract

**Highlights:**

**Public health relevance—How does this work relate to a public health issue?**

- Delayed access to meaningful and professionally relevant work among highly educated migrants is identified as a structural determinant of mental health, social participation, and well-being.
- Prolonged workforce exclusion and credential-recognition delays contribute to psychosocial stress, identity disruption, loneliness, and reduced health-related quality of life among migrants.

**Public health significance—Why is this work of significance to public health?**

- The study demonstrates that integration policies and labor market structures function as upstream public health mechanisms shaping mental health and health equity for migrant populations.
- By linking professional identity loss to well-being outcomes, the findings extend migration health research beyond employment status to emphasize identity, purpose, and social belonging as key health resources.

**Public health implications—What are the key implications or messages for practitioners, policy makers and/or researchers in public health?**

- Public health-oriented integration strategies should prioritize early, meaningful work-based opportunities and faster credential recognition to promote mental health, language acquisition, and social inclusion.
- Researchers and policymakers should incorporate identity continuity and access to professional communities as indicators of successful integration and population health among migrants.

**Abstract:**

**Background**: Highly educated migrants in Norway often experience prolonged delays before accessing employment that matches their qualifications. During this period, many participate in compulsory education or language training, or work in jobs unrelated to their professions. This study explores how the introductory program for migrants and work outside one’s professional field shape health, identity, and the broader integration process. **Methods**: This exploratory qualitative study used semi-structured interviews with eight highly educated migrants enrolled in the introductory program in Kristiansand municipality in Norway. Interviews were transcribed verbatim and analyzed using reflexive thematic analysis informed by Social Identity Theory. **Findings**: Participants described employment, and particularly their former professions, as central to their self-esteem, sense of meaning, and social belonging. Prolonged credential recognition processes and limited opportunities for meaningful social contact due to employment status loss contributed to feelings of stagnation, exclusion, and weakened professional identity. Many participants emphasized a strong desire to contribute to society and regain their professional status. While the introductory program offered valuable peer support and facilitated language learning, it was not experienced as a direct pathway to inclusion in Norwegian society and professional life. The findings indicate that early access to work-based integration opportunities, such as internships or relevant job placements, may enhance well-being, foster language acquisition, and strengthen social inclusion for highly educated migrants. **Conclusions**: The study findings suggest that policymakers and practitioners should prioritize measures that streamline credential recognition and expand early, relevant work-based integration opportunities. Such approaches can improve language development, support identity reconstruction, strengthen social belonging, and ultimately promote better health and integration outcomes for highly educated migrants.

## 1. Introduction

Migration has been established as a social determinant of health [1], with employment playing a crucial role in shaping well-being and social integration in the post-migration phase [2]. Migration often entails various psychosocial stressors, including loss of social networks, uncertainty about legal status, and disrupted professional identity, all of which are associated with elevated risks of depression and anxiety among migrants [3,4]. The recent literature highlights how both professional and social obstacles can compromise migrants’ sense of identity continuity and value in their new host society [5]. In the migration literature, the importance of sense of continuity refers to maintaining aspects of one’s professional and social identity despite migration-related disruptions [6]. Migration will commonly disrupt previous group memberships gained through social and professional networks, removing protective resources and increasing vulnerability to psychological distress [7]. Despite strong qualifications, many migrants face exclusion from professional roles, leading not only to the experience of discrimination but to underemployment with implications for well-being and family stability [8,9].

Identification with the host society is often strengthened through meaningful contact with members of the majority population [10], which supports trust, civic engagement, and social integration [11].

Meaningful activities are understood as a source of purpose that strengthens psychological resilience, even in adverse circumstances [12]. For highly educated migrants navigating prolonged uncertainty and professional stagnation, the literature indicates that activities such as transitional work, language practice, or community participation offer more than routine; they may provide purpose [13].

Migrants, defined as people born outside the country, together with their children, today make up 23.5% of the Norwegian population [14]. In 2024, the employment rate among individuals with an immigration background in Norway was 68%, compared to 79% in the general population [15]. While migrants are often treated as a single category, they obviously represent a highly heterogeneous population. Factors such as country of origin, migration history and status, and broader social determinants of health may influence experiences of employment, integration, and general well-being, with substantial variation both within and across migrant groups in Norway [16].

Furthermore, figures from 2022 show that 40,000 highly educated immigrants were employed in work that did not require their specific professional qualifications [15]. These numbers indicate delayed access to relevant employment and persistent overqualification. Formal recognition of foreign education significantly improves employment prospects and wage levels for migrants [17].

Importantly, meaningful work-related activities may support self-esteem and social belonging even when access to profession-relevant employment is delayed [18]. Hence, engagement in what is experienced as meaningful work could be as important as re-entering a previous profession, even in temporary or permanently disrupted career phases [5].

Highly educated migrants often face a gap between their expectations and the reality of the integration process, leading to what is coined the ‘integration paradox’, where migrants with the highest qualifications report the lowest sense of belonging and satisfaction with integration outcomes [19,20]. Newly arrived migrants often lack access to social networks and spaces that build social capital, increasing the risk of loneliness during the early phase (29). Salway et al. (2020) [21], in a UK-based participatory evidence synthesis, found that interventions creating shared-identity social support groups showed the strongest evidence of positive impact on loneliness. More specifically, shared-identity support groups, where individuals exchange advice, affirmation and purpose, have been found to reduce loneliness [21]. Workplace affiliation may thus serve as a shared-identity setting that reduces loneliness and fosters belonging [22].

Although employment generally promotes health and integration, its positive effects may be limited when migrants are confined to roles that do not reflect their skills, aspirations, or professional identity, potentially leading to underutilization of skills, frustration, and social isolation [23,24]. The concept ‘precarious employment’ is defined as “a type of work characterized by instability, low wages, lack of job security, and adverse working conditions that are often influenced by broader social contexts and individual labor practices” [25]. Precarious employment not only undermines economic security but is also found to negatively affect health and social integration [26]. In contrast, having employment that aligns with one’s qualifications is associated with stronger well-being and a reinforced sense of identity [5]. The inability to use one’s skills undermines identity and well-being, making integration not just a societal challenge, but a public health concern [27].

Previous research has documented the negative health impacts of unemployment and social isolation among migrants [3,4]. However, less is known about how highly educated migrants experience the absence of professionally meaningful work. This gap is particularly relevant for this group, as they often face a mismatch between their qualifications and opportunities available in new host countries.

Understanding how migrants navigate identity, purpose, and well-being in periods of waiting, professional stagnation, or partial participation in work-related activities is crucial for developing more inclusive integration policies.

The present study investigates how highly educated migrants in Norway experience the relationship between identity and work-related activities during the integration process, including participation in compulsory education or work within and outside their original profession. This study contributes to the existing literature by providing an in-depth, qualitative account of how delayed access to professionally relevant work shapes identity and well-being among highly educated migrants in the Norwegian context. While previous research has documented structural barriers to employment, this study highlights the lived experiences of professional underutilization and its implications for identity and social belonging.

### 1.1. The Norwegian Regulatory Bodies for Approval of Foreign Education and Integration Structure

Migrants do not necessarily need recognition of their foreign education to work in Norway; however, it is required for regulated professions. Formal validation of education does improve opportunities in the job market and not least the chances of getting educationally relevant work [28]. Understanding the potential of diverse regulatory bodies to generate both facilitators and barriers for work-related integration is important for examining how highly educated migrants navigate integration and seek meaningful activities while awaiting entry into their professional fields, a central focus of this study.

The Norwegian regulatory framework governs credential recognition and integration programs through the Norwegian Directorate for Higher Education and Skills (HK-dir), which is in charge of assessment and recognition of higher education attained at foreign institutions [28]. HK-dir lists 172 regulated professions requiring recognition of foreign degrees under European Union (EU) directives [29]. While some foreign degrees and professions are automatically recognized, other degrees or professions require an application for recognition and an individual qualifying assessment. As per March 2025, HK-dir reported that they had longer processing times than usual due to a high volume of applications, with a reported estimated processing time of 6-8 months for the recognition of higher education attained abroad [30]. There is, moreover, and more importantly, no guarantee that foreign education will be accepted by HK-Dir, and the reply may contain a conditional acceptance calling for extensive retraining.

As migrants are granted residency in Norway, the Norwegian Directorate of Integration and Diversity (IMDi) decides in which municipality they will live, based on the municipalities’ capacity as well as the migrants’ personal circumstances and relevant work- and educational opportunities. The Law of Integration through Training, Education and Work was designed to strengthen migrants’ participation in society and to increase their opportunities for work to encourage economic independence [31]. Qualifying migrants between the ages of 18 and 55 have both a right and a duty to complete a two-part introductory program with a focus on Norwegian society and language [32], and the program also contains life skills training and components geared at preparing the participant for work or additional education. The Introductory Program has received criticism for causing delays in migrants’ introduction to the labor force [32].‘Praksis’, a hands-on component preparing for work, is included later in the program as an internship to introduce the participants to the Norwegian working environment. It is designed to highlight and strengthen the participant’s competency level within a particular field as well as to strengthen their Norwegian language skills. As detailed by the Norwegian Ministry of Labour and Social Inclusion (AID), the municipality where the migrants settle have the responsibility to arrange such an introductory program within three months of the migrants’ settlement [33]. The participants are financially compensated for participating in the program [31].

### 1.2. Conceptual Framework

This study is informed by Social Identity Theory (SIT), originally developed by Tajfel and Turner [34,35], and aims to explore how professional identity and lack of group belonging influence the well-being of highly educated migrants who are not working within their professional fields post-migration. Social Identity Theory posits that individuals derive part of their self-concept, emotional stability, and sense of belonging from membership in valued social groups, such as professional communities [34,35]. According to the theory, group memberships provide individuals with a shared social identity that contributes to meaning, purpose, and continuity in life, particularly during periods of transition or uncertainty. Haslam et al. explain that, according to SIT, if a group provides a member with stability, meaning, purpose, and direction, it will have a positive effect on mental health [36]. It furthermore suggests that group-memberships increase people’s experience of self-esteem [35]. Migration often disrupts such group affiliations and may lead to psychological distress and professional identity loss. This framework is deemed particularly relevant in the context of highly educated migrants, for whom professional identity may be a key marker of group belonging.

## 2. Methods

### 2.1. Recruitment

This study is a part of the larger project Integration for Health (Int4Health) [37] and particularly addresses Work Package 3 (WP3), which studies integration and health among forced migrants in Norway. WP3 more concretely examines how a work-related intervention affected health and integration among highly educated migrants in Bergen, Norway, with a particular emphasis on migrants with health professional backgrounds. The intervention had a control group in Kristiansand, Norway, that provided quantitative data comparable to the intervention group located in Bergen [38,39]. Prior to the interviews, the interviewer (SKE) introduced herself as a master’s student with an interest in migrant health linked to her personal background of repatriating Expat after living 30 years abroad.

The present sub-study consists of an independent qualitative component linked to the control group, exploring participants’ integration trajectories. Although the broader project includes a focus on migrants with health professional backgrounds, the present sub-study includes participants with diverse professional fields.

A purposive sample of eight highly educated adult migrants was recruited from a larger group of approximately 60 participants enrolled in the Introductory Program in Kristiansand municipality, Norway. Information about the study was shared in both English and Norwegian during a brief presentation at the program site. Interested individuals provided contact details and were later contacted by email. A total of 13 individuals initially expressed interest in participating in the qualitative study. Of these, five did not take part: one withdrew after initially agreeing, two did not respond to follow-up contact, one was unable to participate due to the lack of an available interpreter, and one had provided incorrect contact information. The final sample consisted of eight participants. All participants gave informed written consent and were assured of voluntary and anonymous participation. Each participant received a gift certificate valued at $15 as compensation for their time.

### 2.2. Participants

The eight participants recruited to the study were aged 29–55 and had migrated from Ukraine, Turkey, Zimbabwe, and Mexico due to war, asylum, family reunion, or better opportunities. Although several participants later described their previous professions as meaningful and important sources of identity, migration decisions were often shaped by broader circumstances beyond employment, including an unstable environment, family-related considerations, legal circumstances, and aspirations for future opportunities. Thus, satisfaction with one’s professional role did not necessarily imply satisfaction with broader life circumstances.

They had stayed in Norway between four months and four years. Seven were women, and all held education beyond secondary school as per inclusion criteria in the main study, of which the present sub study is a part, as explained below. Participants were educated in the fields of psychology, medicine, pharmacy, teaching, marketing and IT. At the time of the interviews none of the participants were employed within their professional fields in Norway. Six were awaiting accreditation of their qualifications while two had received accreditation but had not yet found relevant jobs. Six participants were enrolled in the full-time introductory program, while two attended only the language part of the program. Two participants were currently taking part in the internship (paid work placement portion of the program) while two were preparing to start. The interviewer had no prior relationship with any of the participants or the introductory program beyond the initial presentation of the study, and the brief email contact confirming participation.

### 2.3. Data Collection and Setting

This study is an exploratory qualitative interview study using reflexive thematic analysis. The study is informed by a hermeneutic phenomenological perspective, which guided data collection and the interpretation of participants’ experiences.

Social Identity Theory informed both the development of the interview guide, the data collection and the interpretation of the material, guiding our focus to how being outside the workforce during integration processes affects migrants’ professional identity, group membership and ultimately their well-being.

Data were collected by SKE through eight semi-structured interviews conducted in October and December 2023 at the Introductory Program site in Kristiansand, Norway. Some participants were initially hesitant to participate due to language barriers. However, they expressed a strong willingness to share their experiences.

Most interviews lasted approximately one hour and were conducted in English or Norwegian, depending on the participants’ preference. Two interviews were supported by interpreters (one to English, one to Norwegian). One of the interpreters was present on-site for one of the interviews; the other was accomplished with an over-the-phone interpreter. The interviewer has a personal interest in the subject matter due to having been immersed in the migrant experience for 30 years in another country. SKE chose to share this as a personal note before the start of the interview to build trust with the participants. The interviewer adopted a flexible and participant-centered approach. The semi-structured interview guide covered topics related to participants’ sense of belonging, experiences of professional continuity or loss, and the role of work- or education-related activities in shaping identity and health. Open follow-up questions were asked flexibly to encourage the participants to raise issues they considered important and elaborate on their experiences. Reflexive field notes were written after each interview and reviewed at the end of the day. All interviews were digitally recorded with participants’ consent, and the full interviews were transcribed verbatim by SKE. Demographic variables were sometimes adjusted in the transcripts to ensure anonymity. Translated segments were checked for accuracy to ensure fidelity to participants’ meaning. The transcript was returned to one of the participants for review as she had been speaking in length about a personal matter that potentially could identify her. As per her wishes, this segment was excluded from the transcript, which did not affect the results of the study. Participant checking was not conducted.

### 2.4. Data Analysis

Interpretation of the emerging material took place from the very start of the interview process and continued throughout the data collection and write-up phase. Reflexive thematic analysis (TA) guided the post-interview analysis [40]. All interviews were first repeatedly reviewed to ensure deep familiarity with the material. The full material was subsequently inductively coded, manually, by SKE and AB. Initial, similar codes were grouped and refined into themes and sub-themes through an iterative process of review and reorganization. Code and theme development were led by SKE and AB, with peer debriefing and discussion used to challenge interpretations and enhance rigor.

The team discussed data saturation and concluded that no additional interviews were necessary, within the scope of this present study, as no major new insights or themes emerged in the final interviews. Saturation was assessed based on the repetition of themes across interviews, the stability of the coding structure, and the absence of substantially new codes in the later stages of analysis. However, we acknowledge that the heterogeneity of the sample may limit claims to data saturation in a conventional sense, as the study was not designed to compare subgroup differences but rather to identify shared patterns across participants.

The all-female research team included both Norwegian and migrant-background scholars with experience in qualitative research and migration studies. At the time of data collection, the research team consisted of SKE (master’s student), KYA (PhD candidate), and ED and AB (professors).

Reflexivity was practiced throughout the research process; team members regularly discussed their own positionality, both as insiders and outsiders to participants’ experiences, and how this could influence data collection and interpretation.

## 3. Findings

Despite the study participants holding higher education degrees and most having substantial work experience from their countries of origin, all the participants faced prolonged delays in entering professionally aligned roles in Norway, and none of them were at the time of the interviews employed in positions relevant to their previous education or professional background.

All the participants expressed a strong desire to enter working life to contribute to society through work and emphasized the personal significance of employment. They described feelings of extended waiting, starting over, or feeling lost, alongside a marked lack of social connection with the local community. All the participants shared a longing to return to work. While participants acknowledged financial independence as important, their narratives primarily framed work in terms of a source of identity, purpose, and social contribution rather than solely an economic necessity. The following sections explore the core themes that emerged during the interviews: The importance of professional employment, The emotional toll of credential or residential limbos and Social isolation from the Norwegian society. Quotations from the participants’ narratives are assigned numbers.

### 3.1. Theme 1: The Importance of Professional Employment

Two sub-themes emerged in relation to how participants perceived the importance of professional employment in a migration context of being unable to work within their field. The themes reflect how participants described professional work as central to their sense of meaning and identity:

The significance of work and The importance of making a useful contribution.

#### 3.1.1. The Significance of Work

Participants consistently described work as central to their self-esteem, pride, and sense of belonging. Many described their profession not simply as a job, but as a core part of their identity. As one participant reflected: “I think there was a time that my work was not only part of my life, but it was my life” (P5). She continued as follows:

In my culture, it’s very common that society pushes you to work a lot, and to have an education and find a good job. And there is a time when your job becomes your life. I think that’s why, for me, having this cultural background, a job is one of the most important pillars of my life (P5).

Another participant shared the emotional dimension of her profession: “In the past, [teaching] was so many things. It was everything for me. But now, I don’t feel anything. I was known for it [being a good teacher]” (P6). Even those who spoke somewhat more pragmatically about work expressed a longing to return to their original professions: “It would be great to get back into my profession. I know it well. I miss my work” (P7). The latter participant nevertheless had good experiences with her internship, which was unrelated to her previous profession. Asked how her internship affected how she was doing, she exclaimed, “I feel better, working at an internship here! I feel better, I am happy! […] I started feeling calmer and more confident. I noticed changes” (P7).

#### 3.1.2. The Importance of Making a Useful Contribution

The desire to return to work was less focused on financial independence than on the wish to regain a sense of usefulness, recognition and contribution. Several participants expressed guilt or frustration about not being able to contribute to the society of which they were a part. The study participants expressed this in slightly different ways saying for example, “I like to feel useful. That I can do something” (P1), “Sometimes I feel guilty of not having a job” (P5), or one of the participants summed up the importance of feeling useful and contributing by working, explaining:

When I do my job, it’s with pleasure. It means a lot to me, and I want to be useful to people. […] I would like to start working as soon as possible within my profession and be useful to this country (P8).

Another participant described how her years as a teacher and PhD-level researcher in her home country made her work feel meaningful and fostered a sense of belonging in academia. She described how she felt a sense of pride when she presented valuable or important research because it provided a “benefit to society” and expressed why she hoped to continue working as a teacher and researcher in Norway: “It is the way I can feel like myself, here. Because I really feel most like myself when I am a part of the professional community” (P2).

### 3.2. Theme 2: The Emotional Toll of Credential or Residential Limbos

Participants expressed that they felt anxious while waiting for immigration decisions or credential recognition. They alluded to their lives being put on hold and expressed frustration about being told only to wait your turn when seeking updates. Some felt as though they were forced to start their careers all over again as they discovered during the waiting process that their qualifications might have limited value in a Norwegian context. The substantial barriers experienced in gaining accreditation for former education consequently forced some of the participants to reconsider former career trajectories. One shared the following:

I also thought about, if it is so difficult to prove my doctor’s diploma; I also have experience teaching at a medical university. And I have a Ph.D., so I think maybe this can be useful here. (P3).

While waiting for credential recognition or residency, many experienced prolonged uncertainty and professional stagnation. Some of the participants who had been in Norway for longer than a year without gaining access to jobs aligned with their qualifications expressed concern that they might never again be able to work within their original profession. Without a workplace community, participants simultaneously had fewer opportunities to practice the Norwegian language and to gain access to social arenas to build new friendships.

When asked in what ways their experience of not having a job at this time might be affecting their health or how they were doing, one of the participants shared that “I feel like I am not of any use for anything. Our objective should be that migrants have good mental health because people are harmed by the waiting period” (P8). Having had to put her PhD thesis on hold during the migration process, another career professional described her experience as follows: “Sometimes I feel lost, like I lost some parts of myself in [my home country]. Maybe it is my studies that I left [behind]” (P2). Further describing how postponing her Doctoral research and remaining outside the workforce was experienced as a sense of demotion to a school-age child, she expressed how it affected her wellbeing: “Sometimes my heart is aching, […] and sometimes I feel really depressed” (P2). Another participant became emotional as she similarly conveyed her sense of loss of identity as she missed a working community: “I almost feel lost—like I am unsure of who I am!” (P5). While motivated to achieve a higher degree of education to further her chances of employment, she explained that she still struggled with the experience of losing her status as a professional within her field:

If you are a student again, these things come to mind that, okay, now you have to start [over] again. And then comes the process of finding a job, like it’s your first time working. And yeah, it’s hard, and it really affects me, not having a job now (P5).

One participant had been waiting for a residency decision for the past 11 months. She was attempting to finish writing her PhD thesis while simultaneously attending the Introductory Program and described how her current and temporary living-situation at an asylum reception center for migrants was affecting her:

What is actually affecting me, is that I haven’t had an interview with UDI (The Norwegian Directorate of Immigration) for such a long time. Meaning, [I am dealing with] the unknown, and the fact that I am living in shared accommodations now.—That I am [living with] people from many different cultures, and I am trying to write my dissertation even though I don’t have a desk. And I don’t know how long this will last. This is what is affecting me the most. And when I try to find a solution, for example, when I call the Directorate of Immigration… [the response is, Participant quoting UDI:] “You have to wait. You have to wait your turn.” That’s the only thing they’re saying. This is very tiresome. (P8)

In contrast, one of the study participants had recently received a positive notification on the application for a residence permit. Although she was currently stagnant professionally, she nevertheless expressed newfound relief at finally being able to move forward with her plans due to the upcoming resettlement in a named municipality. With her residency in place, she explained that she would be able to enroll at a local university to supplement her existing degree and eventually continue working within her profession. She shared her excitement about her upcoming move, saying,” I’m so happy for knowing the exact place and exact time, because I know the path forward. Uncertainty makes people anxious” (P2).

### 3.3. Theme 3: Social Isolation from Norwegian Society

While Theme 2 focused on experiences of professional exclusion, participants also described a sense of broader social disconnect from the Norwegian society when remaining outside work life. Many struggled to form relationships with Norwegians outside the context of the Introductory Program, citing language barriers and cultural differences as key ingredients. As one participant who had arrived five months earlier observed, “There is not much interaction between Norwegian people and the others. It is like a cultural thing; it is not like in [my home country]” (P6). Another participant, who had been in the country for four years, shared the same observation: “I don’t socialize as much. It comes from [having a] language barrier. The loneliness here, it’s a bit much. It gets you” (P4).

Although participants generally valued the Introductory Program, they perceived it as insufficient for fostering broader societal inclusion. Several emphasized the importance of work as a space for both social and community engagement, and as one explained, “It’s good not to be home all the time. If it’s a good workplace, good people, then it’s good [socially] also” (P1). Another participant expressed the socialization dimension of work very directly, saying, “Work is essential. It is socialization in society” (P7). One participant recollected the social dimension of work from former life experience:

I was very friendly with my friends in the workplace. We had many activities, like lunch, or we did other things together. […] A person from [my country] doesn’t like to be alone; we love to be in a group. Interaction is important (P6).

Most of the study participants socialized primarily with other participants in the Introductory program. However, opportunities to build friendships and thus practice the language skills, especially with Norwegian-born individuals, were found to be very limited. As one participant noted: “People are very busy with their lives here in Norway. No-one has time to come for tea at your home” (P4).

One woman who initially felt strongly affected by social isolation stressed how learning the language eased the sense of being an outsider. She described her experience of progress in language acquisition as being transformative for being alive and feeling that you are part of the surrounding society: “Now I am relaxed, and I’m comfortable because I have started to understand the language, and it makes me a real human being; a living creature in the world, in society” (P2).

Being outside the workforce implied spending more time alone. In a Norwegian context, loneliness was said to be exacerbated during the winter months when the environment was cold or dark. The darkness and cold weather were explained to affect people’s moods and lifestyles, including how they would choose to stay indoors if they did not have concrete business outside the home. As described by one participant:

Of course, there’s rain in my home country, but not like here. I think it’s really different, like the darkness for example, during winter. Last winter was really bad. Sometimes it’s difficult with the weather. It really impacts me (P5).

Another shared her experience with winter depression:

I remember we did a paper on winter depression [in my educational program]. It was really interesting. I think I might have had it, winter depression. I think that’s the one thing that was a problem for me: it gets quiet, really quiet, and it gets really dark. Yeah. For someone coming from a loud country, very sunny, it was taking a lot of adjustment. (P4).

Participants appreciated the Introductory Program for offering language training and a shared-identity space where they could form friendships with others who understood their experiences and could provide emotional support. However, as we have seen in this section, these valuable social connections were confined to fellow migrants and did not extend to members of the broader Norwegian community, leaving participants feeling socially isolated beyond the program. While the program offered a safe space for bonding, these bonds were perceived as insufficient for achieving societal integration in the larger Norwegian society and for restoring professional identity.

These observations highlight that while shared-identity spaces facilitated by the Introductory Program foster friendships and emotional support, they do not make up for the deeper sense of lack of societal belonging that is generated through professional networks at a workplace and that fosters contact with the majority population and their broader societal surroundings.

## 4. Discussion

This study explored how highly educated migrants, while engaged in the Introductory Program, experienced work-related activities as particularly meaningful. At the same time, being outside the labor market made them feel disconnected from everyday life in Norway. Prolonged exclusion from work affected both their sense of professional purpose and their opportunities for social connection [41].

While the concepts of ‘mobility dissonance’ and ‘Ulysses Syndrome’ did not inform the study design, data collection, or analytical approach, they are drawn upon in the discussion section to help contextualize the findings within the broader literature on migration, integration, and psychosocial well-being.

Participants’ narratives pointed to a disruption of an ‘identity cluster’, referring to the interconnected sense of continuity, belonging, and well-being. This aligns with previous research documenting identity loss in post-migration contexts [36,42,43,44]. Work consistently emerged as a lifeline in the participants’ narration, symbolizing a potential pathway to restore purpose in their lives. Participants described employment not primarily as a source of income, but as a desired activity that provided purpose, recognition and a sense of belonging. In our material, employment also seemed to offer a way to reconnect with aspects of the participants’ professional selves. Without work, participants described feeling “lost,” and viewed employment as a key avenue to regain social recognition, experience inclusion, and contribute meaningfully to the larger society. Prolonged bureaucratic delays, especially related to credential recognition and residency processing, disrupted professional continuity and prevented them from accessing work-related social environments. Participants expressed in various ways that this contributed to feelings of stagnation, uncertainty, and emotional distress.

While the link between employment, identity, and well-being is not unique to migrants, the present study suggests that these challenges may be intensified in a migration context due to disrupted professional networks, language and cultural barriers, barriers to credential recognition, and limited access to relevant employment.

These study findings seem to be examples of Social Identity Theory’s central claim that group membership and identity continuity are critical for psychological well-being [45]. Participants’ reflections illustrated how their previous professional roles had provided them with both a sense of mastery and a sense of belonging, forming a key part of their identity. Migration disrupted this continuity, and unemployment or underemployment led to feelings of inadequacy and exclusion. Importantly, participants longed for recognition from a relevant Norwegian professional community; a new, aspirational in-group that they perceived as difficult to access. This highlights a core SIT dynamic; when valued group membership is blocked, individuals experience identity threat and psychological strain [46]. The longing for validation as competent professionals illustrates migrants’ attempts to achieve a sense of identity continuity as members of a professional in-group within the host society, even when full membership in the society remains out of reach [47]. While Social Identity Theory provides a useful lens for enhancing the understanding of identity and belonging, its focus on group processes may underemphasize the role of migration policies, legal status, and labor market barriers, which are central to the very real structural constraints that shape migrants’ lived experiences of actual opportunities.

The experiences encountered among the present study’s participants moreover echo Bygnes’ (2019) concept of ‘mobility dissonance’, which points to post-migration work-related stagnation caused by extensive and drawn-out bureaucratic processes, in Bygnes’ study experienced among Syrian migrants in Norway [48]. Our participants’ accounts of waiting for credential recognition, similarly reflect the dissonance between what they long for and the increasing recognition that what you aim for may not be realized.

Prolonged waiting times for residency, credential recognition, and job market entry have in several studies been shown to have negative impact on migrants’ mental health and thus the potential for social integration [24,25,26]. Such stagnation, moreover, resonates with the concept of Ulysses Syndrome [49], which refers to the cumulative stress and grief linked to multiple migration-related losses, including professional identity. For highly educated migrants, the inability to become employed within their field of expertise represents a profound “working-loss,” compounding other losses such as social networks, language and socio-cultural familiarity. These layered stressors can lead to feelings of demotion or devaluation, uselessness, and identity fragmentation [50]. In Norway, residency processing times vary by migrant category and origin, with delays reportedly worsening in 2024 due to an increasing number of applications. According to the Norwegian Organization for Asylum Seekers (NOAS), waiting times for asylum interviews more than doubled from 2021 to 2024, increasing uncertainty and distress for the ones waiting in line [51].

Participants described in various ways how their lives were put on hold. They used phrases like how they were forced to “wait for your turn” and “to start again”, echoing the frustration of Bygnes’ participants [48]. In Bygnes’ study, highly educated Syrian migrants mobilized their ‘classed’ resources, including their educational background, as a source of continuity through the post-migration period. These resources did not remove experiences of stagnation, but they offered a partial buffer during the early years after migration. In contrast to Bygnes’ findings, our participants appeared to experience both occupational and social marginalization, limiting their ability to draw on established educational or class resources (their education) as a source of continuity.

SIT helps make sense of the dynamics at work; when migrants are excluded from their original professional communities, they lose an important identity anchor. Opportunities to reconnect with their professional groups or gain access to related professional pathways are in such circumstances highly likely to establish a sense of meaning and continuity at a time when many feel anxious and lost [34]. Salway et al. [21] emphasize the importance of shared-identity social groups for emotional and informational support, as well as for affirmation, in terms of validation of the participants’ competence and value [21]. Interventions that provide meaningful work-related activities may thus mitigate the identity disruption and promote health by restoring a sense of purpose and belonging among migrants [18]. In our study, the Introductory Program was experienced as important for language learning and peer support, offering partial relief from isolation. However, it did not restore professional identity, nor did it provide access to Norwegian networks, underscoring the need for work-based and other integration-related opportunities.

Internships (‘praksis’) and other work-based elements of the Introductory Program were highly valued for providing authentic opportunities to meet Norwegians, practice the Norwegian language in natural contexts, and to build confidence or self-esteem, while also for some, offering a sense of continuity by preserving aspects of their professional and social identity. While the Introductory Program provided a shared-identity group that fostered friendships and reduced loneliness, participants emphasized that discipline-related belonging—such as reconnecting with professional peers—was qualitatively different. Professional affiliation would offer not only social support but also validation of competence and ensure identity continuity, dimensions which the program could not provide. This distinction underscores why combining educational components with relevant practical work placements emerges as critical for restoring identity [52] and well-being [7].

These findings suggest that participants’ experiences are shaped by an interaction between identity-related processes and structural factors, including labor market barriers, credential recognition systems, and integration policies. A study from Denmark similarly demonstrates how early access to relevant work placements and opportunities for language practice with native speakers may help speed up integration in a post-migration phase [53].

While Social Identity Theory provides a useful lens for understanding identity and belonging, its focus on group processes may underemphasize the role of structural constraints, such as migration policies, legal status, and labor market barriers, which are central to migrants’ lived experiences.

### 4.1. Policy Recommendations

Even though EU integration metrics focus primarily on employment rates and language proficiency [54], our findings suggest that, from a policy perspective, identity continuity and professional reintegration should be considered key indicators of successful integration. This aligns with calls from scholars advocating for more holistic integration frameworks in host countries [55].

Our findings highlight the importance of recognizing prior qualifications as a central component of professional reintegration and identity continuity [17]. In addition, they point to the need to reduce bureaucratic delays and introduce work-based learning earlier in the integration process. Policy measures should therefore prioritize early access to professional pathways, such as internships in relevant fields, alongside tailored language support embedded in workplace settings. Furthermore, structured opportunities for contact with Norwegian-born individuals are likely to foster belonging and reduce isolation, especially for migrants outside formal employment.

### 4.2. Strengths and Limitations

The study contributes to field of migration health research by illustrating how the absence of meaningful, work-related activity during the integration process can undermine well-being and foster experience of social exclusion among highly educated migrants in Norway.

The study’s small, predominantly female sample and single-site design reduces transferability. The heterogeneity of participants makes it impossible to explore subgroup differences related to migration status, country of origin, and profession. The predominance of female participants may also have influenced how experiences of work, identity, and integration emerged in the material. Consequently, the findings should be interpreted as exploratory and illustrative, providing insight into shared experiences of professional underutilization and its impact on identity and well-being among highly educated migrants. The sample may be subject to self-selection bias; individuals who chose to participate may potentially have been more engaged in the integration program or more motivated to share their experiences, which may influence the perspectives captured in the data. Two interviews were conducted with the support of interpreters, which may have influenced both data generation and interpretation. The presence of an interpreter can affect the flow of communication and the level of rapport established between interviewer and participant, potentially shaping how experiences were expressed. Additionally, translation processes inherently involve interpretation, and subtle meanings, or culturally embedded expressions, may be modified or lost in the translation process. Although the interviews were transcribed in their entirety and translated sections were checked for accuracy, some nuances may have been lost. This could have implications for the interpretation of participants’ narratives and the development of themes.

We believe the findings nonetheless offer valuable insights for comparable migrant populations who are navigating credentialing and integration processes. Researcher positionality was acknowledged and mitigated through team reflexivity, supporting balanced interpretation and analytical rigor despite the study’s exploratory scope.

## 5. Conclusions

This study suggests that restoring a sense of social identity through meaningful, work-related activity may play a vital role in the well-being and integration of highly educated migrants in Norway. Delays in workforce entry and credential recognition appear to limit opportunities to maintain professional identity and social belonging, which may contribute to experiences of disconnection. Based on the material, we argue that early access to work-related activities and recognition of prior qualifications may support both integration processes and migrants’ well-being. By providing qualitative insight into how highly educated migrants experience professional underutilization, this study highlights the importance of identity, purpose, and social belonging as key dimensions of health and integration. Future research should further explore how work-related integration processes unfold across different migrant groups, including potential gender and migration-related differences. Additionally, it should examine the long-term impact of diverse aspects of integration policies through longitudinal and comparative studies. Although this study is situated in the Norwegian context, the findings may have relevance for other high-income countries with similar educational, employment, and integration systems, where highly educated migrants face challenges related to credential recognition and access to relevant employment.

## Data Availability

The datasets analyzed during the current study are not publicly available due to the privacy of the participants but are available from the corresponding authors on reasonable request.

## Abbreviations

The following abbreviations are used in this manuscript:

AID	Norwegian Ministry of Labour and Social Inclusion
EU	European Union
HK-dir	Norwegian Directorate for Higher Education and Skills
IMDi	Norwegian Directorate of Integration and Diversity
NOAS	Norwegian Organization for Asylum Seekers
SIT	Social Identity Theory
TA	Thematic Analysis
UDI	The Norwegian Directorate of Immigration
